# Challenging the heterogeneity of disease presentation in malignant melanoma—impact on patient treatment

**DOI:** 10.1007/s10565-018-9446-9

**Published:** 2018-10-24

**Authors:** A. Marcell Szasz, Johan Malm, Melinda Rezeli, Yutaka Sugihara, Lazaro H. Betancourt, Daniel Rivas, Balázs Gyorffy, György Marko-Varga

**Affiliations:** 1 Center of Excellence in Biological and Medical Mass Spectrometry, Lund University, BMC D13, 221 84 Lund, Sweden; 2 Division of Oncology and Pathology, Department of Clinical Sciences Lund, Lund University, 221 85 Lund, Sweden; 3 Cancer Center, Semmelweis University, Budapest, 1083 Hungary; 4 MTA-TTK Momentum Oncology Biomarker Research Group, Hungarian Academy of Sciences, Budapest, 1117 Hungary; 5 Department of Oncology, Lund University, Skåne University Hospital, 221 85 Lund, Sweden; 6 Department of Translational Medicine, Section for Clinical Chemistry, Lund University, Skåne University Hospital Malmö, 205 02 Malmö, Sweden; 7 Clinical Protein Science and Imaging, Department of Biomedical Engineering, Lund University, BMC D13, 221 84 Lund, Sweden; 8 Institute of Environmental Sciences and Water Research, IDAEA, Spanish Research Council (CSIC), Barcelona, Spain; 9 2nd Department of Pediatrics, Semmelweis University, Budapest, 1094 Hungary; 10 Division of Life Science and Biotechnology, Yonsei University, Soel, Korea

**Keywords:** Melanoma cancer, Tumor heterogeneity, Proteomics, Mutation, Mass spectrometry imaging

## Abstract

There is an increasing global interest to support research areas that can assist in understanding disease and improving patient care. The National Cancer Institute (NIH) has identified precision medicine-based approaches as key research strategies to expedite advances in cancer research. The Cancer Moonshot program (https://www.cancer.gov/research/key-initiatives/moonshot-cancer-initiative) is the largest cancer program of all time, and has been launched to accelerate cancer research that aims to increase the availability of therapies to more patients and, ultimately, to eradicate cancer. Mass spectrometry-based proteomics has been extensively used to study the molecular mechanisms of cancer, to define molecular subtypes of tumors, to map cancer-associated protein interaction networks and post-translational modifications, and to aid in the development of new therapeutics and new diagnostic and prognostic tests. To establish the basis for our melanoma studies, we have established the Southern Sweden Malignant Melanoma Biobank. Tissues collected over many years have been accurately characterized with respect to the tumor and patient information. The extreme variability displayed in the protein profiles and the detection of missense mutations has confirmed the complexity and heterogeneity of the disease. It is envisaged that the combined analysis of clinical, histological, and proteomic data will provide patients with a more personalized medical treatment. With respect to disease presentation, targeted treatment and medical mass spectrometry analysis and imaging, this overview report will outline and summarize the current achievements and status within malignant melanoma. We present data generated by our cancer research center in Lund, Sweden, where we have built extensive capabilities in biobanking, proteogenomics, and patient treatments over an extensive time period.

## Introduction

Healthcare is expensive and healthcare costs are steadily on the rise in most countries. New drugs are expensive as are many of the newly emerging diagnostic tests. Precision medicine, however, may aid in reducing the cost of patient care and can be readily available for all patients regardless of social standing. Early investment in precision medicine measures can be financially beneficial in the long term, while at the same time increasing the quality of life for patients, and also has the potential of extending the life expectancy with better life quality.

When an oncologist decides to prescribe chemotherapy or not, the decision is often based on the stage of the cancer. In contrast to patients with a more advanced disease, early stage patients are usually not given chemotherapy, as they tend to have a good prognosis. This strategy is believed to save money and resources, and limit unpleasant side effects to the patients in both the short term and long term. It is often observed, however, that the early stage cancers relapse and the patient eventually receives chemotherapy treatment, albeit often too late. Genetic tests emerged to save even more on chemotherapy in selected clinicopathological groups of patients with equivocal outcome, where the prognosis can be predicted by molecular tests (Paik et al. 2004; Sparano et al. 2015).

Even when a drug is given to the “right” patient, adverse drug reactions ranging from mild to lethal can occur. Many adverse drug reactions are due to variations in drug metabolizing proteins, i.e., variations that affect the response of an individual to a drug. In the USA alone, the cost of adverse drug reactions in 2013 was estimated at more than 30 billion USD (Sultana et al. 2013). The situation is similar in Europe. Adverse drug reactions are associated with substantial morbidity and mortality (European Commission. Proposal for a regulation amendment concerning pharmacovigilance of medicinal products for human use. Regulation (EC) No 726/2004. Impact assessment. 2008. Available at http://ec.europa.eu/health/files/pharmacos/pharmpack_12_2008/pharmacovigilance-ia-vol1_en.pdf. Accessed 3 Sept 2014). Throughout the EU, approximately 5% of all hospital admissions and 197,000 annual deaths have been estimated as a consequence of adverse drug reactions.

Precision medicine is expected to be implemented in many areas of routine healthcare. One of the most important areas where it will become the foundation of future cancer therapeutics is in cancer diagnostics and treatment. Now used in many countries, one of the best-known examples of precision medicine is the treatment of certain lung, breast, and other cancers with gefitinib and erlotinib. Both drugs are tyrosine kinase inhibitors (TKIs) of the epidermal growth factor receptor (EGFR). These drugs are only effective in cancers with mutated and overactive EGFR expression. These mutations confer increased sensitivity to TKIs such as gefitinib and erlotinib. Diagnostic tests to detect EGFR mutations are often performed prior to treatment to aid in predicting which patients will most likely respond to therapy with, e.g., gefitinib/erlotinib. When a cancer patient no longer responds to these targeted agents, another TKI can be administered, e.g., osimertinib. Once a companion test for the mutation has been performed and the mutation detected, the patient can be further treated with the appropriate TKI.

TKIs are excellent examples of precision medicine and are fundamentally changing the way new diagnostics and treatments are expected to evolve in future healthcare. Expanding the value of diagnosis by biomarker development and optimized treatment is the key to providing an overall increase in efficacy and safety to cancer patients. Biomarkers have been classified into three categories: (a) POM, (b) POP, and (c) POC and are defined as follows:

Biomarkers for “Proof of Mechanism”—POM:

A biomarker demonstrates an effect, which results in a functional change related to the proposed mechanism-of-action. The proof of mechanism effects can be measured with, e.g., an in vivo assay, where an effect is measured following an appropriate stimulus.

Biomarkers for “Proof of Principle”—POP:

A biomarker demonstrates an effect that results in a biological change that is closely related to the proposed mechanism-of-action and known to be associated with disease activity in patients. The proof-of-principle biomarker read out is proven in a dedicated patient study. It can be a measure of, e.g., an acute phase marker regulation in patient studies after drug intervention.

Biomarkers for “Proof of Concept”—POC:

The biomarkers used in clinical studies, which relate to the proof-of-concept will measure a study end point that demonstrates an effect on a clinical end point. Proof-of-concept biomarker evaluation must be performed in patients with the disease in question. In cancer studies, a tumor reduction would be a positive effect where the biomarker quantitation provides an additional positive effect. These biomarker categories are used within drug discovery, drug development, and the clinical field.

The National Cancer Institute (NCI) has identified precision medicine-based approaches as key research strategies to expedite advances in cancer research and precision medicine. This concept is the cornerstone of the Cancer Moonshot program. Championed by the 47th Vice President Joe Biden, the program is a major effort to move cancer patients towards better treatment and care in the next 5 years. The Cancer Moonshot program was launched to “accelerate cancer research aims that make more therapies available to more patients, while also improving our ability to prevent cancer and detect it at an early stage” (https://bidencancer.org*/*, https://www.cancer.gov/research/key-initiatives/moonshot-cancer-initiative). Ultimately, the mission is to eradicate cancer. To date, ten countries, including Sweden, have joined the Cancer Moonshot program to further strengthen the combined research activities. At the Cancer Center in Lund, Sweden, we have built a cutting edge capability, including biobank archives, fully automated with robotic processing as well as proteogenomics, and patient treatment protocols capturing clinical data and disease progressions (Malm et al. 2018; Sugihara et al. 2018).

## Cancer impact by optimal treatment

In 2014, there were an estimated 14.7 million people living with cancer in the USA. Based on 2012–2014 data (Noone et al. 2018), approximately 38.5% of men and women will be diagnosed with cancer at some point in their lifetime. Based on 2010–2014 age-adjusted cases and deaths, the number of new cases of cancer was 442.7 per 100,000 men and women per year and the number of deaths were 166.1 per 100,000 men and women per year (Noone et al. 2018). Although the 5-year relative survival by year of diagnosis between 1975 and 2013 increased from 48.9 to 69.2%, there is still room for improvement in the field of cancer research. Increase in number of cases and better outcomes are partially due to better screening methods, which detect more cases but also in earlier stages.

Worldwide, there is an increasing interest and need to support research areas that can assist in improving disease understanding and advancing patient care. This includes novel medicines such as “precision medicine”, alternative treatment technologies, and early indication of disease diagnosis utilizing both imaging techniques and biomarker diagnostics (Price et al. 2009). Ultimately, it is the patients who are suffering and experiencing the limitations of treatment today.

Due to an ever-increasing number of cancer patient cases, there is a considerable shift in the future demand and expectations of the healthcare systems. Today, every third Swedish inhabitant will experience a cancerous disease during their lifetime. This creates novel opportunities and challenges for the medical research community to drive patient-centric and technology-driven research strategies to improve overall patient care. This becomes an ever-increasing challenge for modern healthcare.

These new developments need to occur now. Due to an increasing cost to society, and increasing suffering and pain for the patients, cancerous diseases are major target areas within the healthcare system.

## Melanoma disease biology

**Fig. 1:**
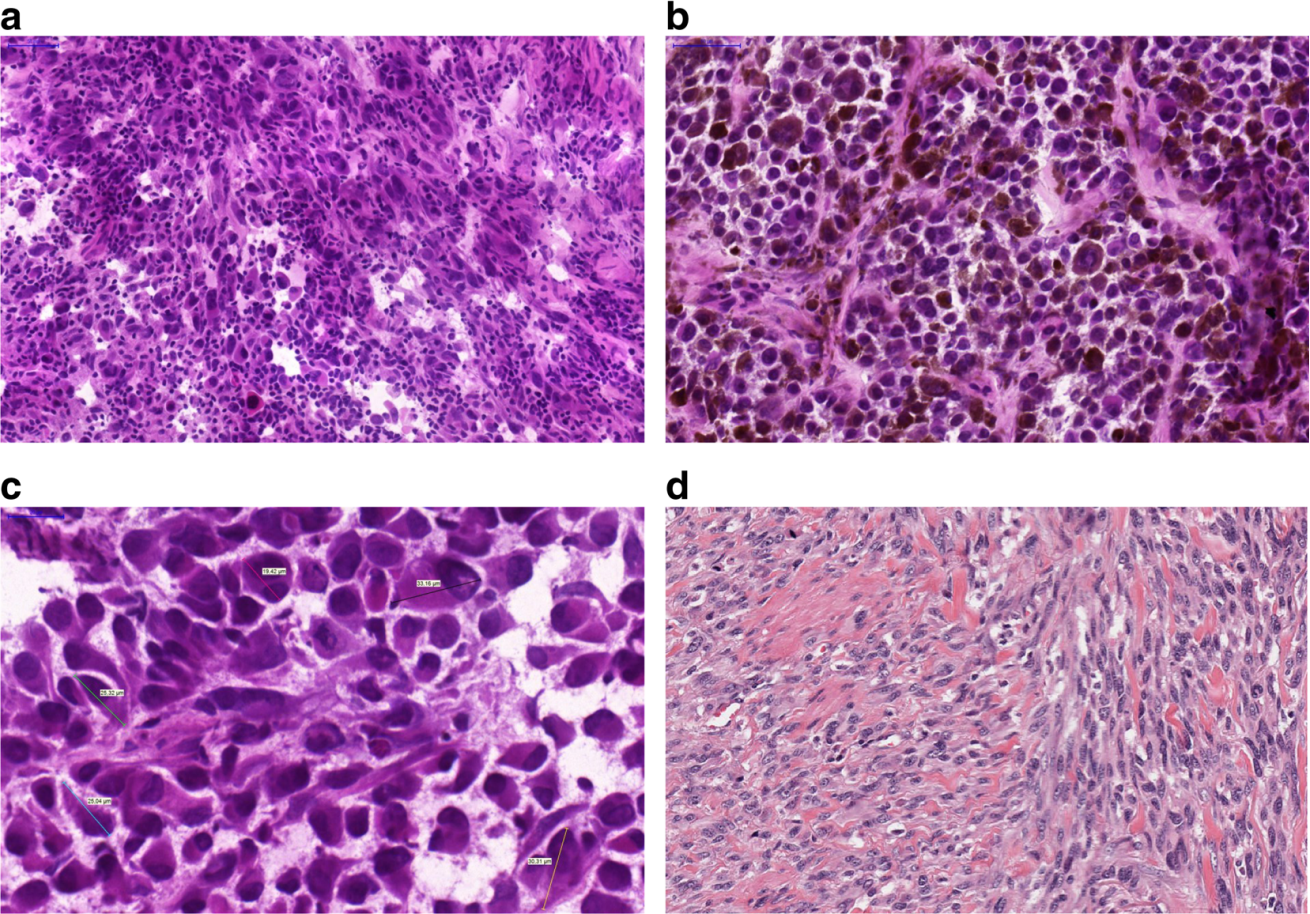
Histological appearance of melanomas. **a** A non-pigmented tumor composed of malignant melanocytes infiltrated by lymphocytes **b** A pigmented tumor producing melanin mostly composed of epithelioid shaped cells (10 ×). **c** Variable sizes of tumor cells are noted in a tumor; multinucleated cells can also be identified. **d** Spindle cell melanoma displaying fascicules of elongated melanocytes

With respect to variation in clinical symptoms, appearance, and eventual biology in patients plus the morphological and molecular variation in an individual tumor, malignant melanoma is a heterogeneous disease (Fig. 1).

**Fig. 2:**
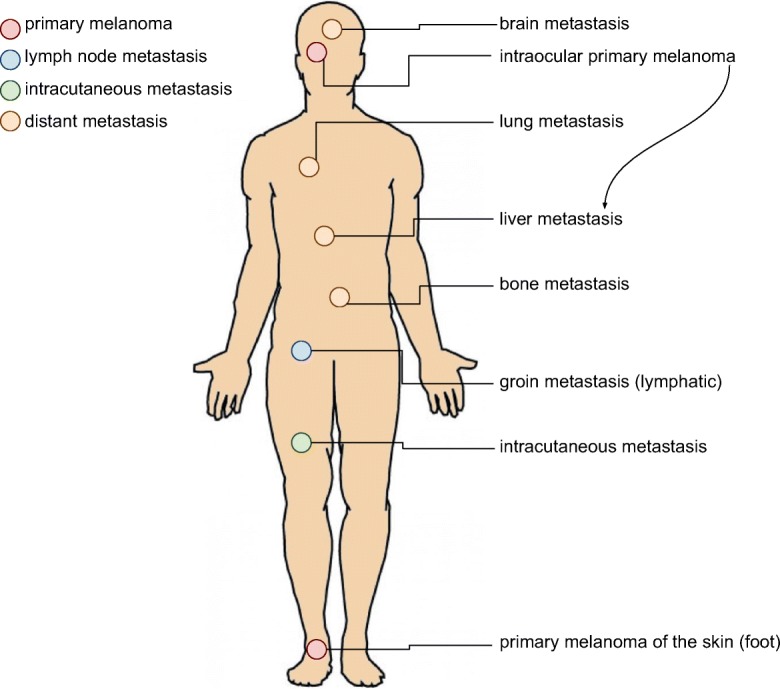
Progression of melanoma depicting the most common sites of metastasis development: primary tumor of the foot; lymphatic spread into the groin; and hematogenous spread to lung, liver, brain, bones, or skin. Malignant melanoma can essentially develop metastases anywhere in the body. Note, intraocular melanomas often give rise to liver metastases

Nevertheless, tumor progression is still mostly related to initial clinical-pathological properties, and the stage of the melanoma. These tumors develop metastases at any location at any time, involving both the lymphatic system and distant organs (Fig. 2). At the morphological and molecular level, the inherent heterogeneity of a tumor can be the cause behind the behavior of a given malignant disease (Sugihara et al. 2014; Welinder et al. 2014). Depending on the sample heterogeneity, a diagnosis may or may not be revealed at the histopathological or at the molecular levels. The technical properties of a diagnostic test, e.g., next generation sequencing coverage can be responsible for diagnosing a mutation spot in the minority of the cells examined. When a metastasis is discovered in a patient with unknown primary, however, melanoma must be included in the differential diagnostic list until confirmation or exclusion by routine pathological experiments including protein level studies, e.g., immunohistochemistry. A spindle cell lesion negative for HMB-45 and Melan-A stains, but displaying S-100 positivity with a clinical history of primary desmoplastic melanoma later disclosed, is a classic pitfall in pathology. Or an intraocular melanoma, e.g., is considered to develop liver metastasis following progression, for which the biological reason has yet to be discovered.

In addition to diagnostics and clinical-pathological classification into superficial spreading, nodular, lentigo maligna, and acrolentiginous melanomas (Mooi and Krausz 2007), recent molecular diagnostics can delineate subtypes of melanoma (possessing mutations in BRAF, NF1, RAS, or triple wild type) (The Cancer Genome Atlas Network 2015). Currently, sequencing studies of stage 4 disease are performed in a stepwise fashion. Firstly, the BRAF status is determined; then, the RAS status, then NF1, and c-kit mutations are considered for acrolentiginous melanomas. As we envisaged, these former mutations are mutually exclusive. If one was positive, the rest were not routinely screened (Platz et al. 2008). Exceptions arose in such a dynamic fashion that the next generation of diagnostics will assess all these genes (and many more) for a possible pathologic change (Chiappetta et al. 2015; Thomas et al. 2015). Heterogeneity at the cellular and consecutively at the molecular level might be present in this setting and can give answers when the mutational spectrum and tumor biology are investigated (Welinder et al. 2013; Yakovleva et al. 2015).

To establish the basis for our melanoma studies, we created the Southern Sweden Malignant Melanoma Biobank, which contains a large collection of tissues and blood samples with accurately characterized tumors and patient information (Welinder et al. 2013). We have investigated and discovered previously undescribed proteins and sequences in malignant melanoma lymph-node metastases (Welinder et al. 2015). Next, we examined ten pilot cases from the perspective of tumor composition: stepwise sectioning was applied to the histopathological and proteomic investigation by mass spectrometry (Welinder et al. 2017). Utilizing the versatility of high-quality proteomic data supplemented with functional annotation (Database for Annotation, Visualization and Integrated Discovery (DAVID)) and pathway analysis (Ingenuity Pathway Analysis (IPA)), we focused on relating high-resolution proteomic data to histopathological evaluation of the tumor samples and patient survival information. Several proteins were identified that positively correlated to tumor tissue content and upstream regulators. HEXB, PKM, and GPNMB were proteins that were identified a significantly related to clinical outcome. These could therefore play a role in the process of progression from disease stage 3 to stage 4 and poorer outcome (Welinder et al. 2017).

Heterogeneity at the genetic level also has a major impact in melanoma, and quantitative and qualitative studies have emerged. For instance, a minor population of cancer cells in each tumor may undergo mutation and give rise to a cohort of cancer cells that possess a mutational pattern different to the other cells. If that mutation can be addressed or has therapeutic consequences, it is crucial to identify and locate such mutations, which have sensitivity issues to solve. With the advent of newer and more sensitive detection methods, a low incidence of a mutation (e.g., a partially BRAF V600E-mutated malignant melanoma) can be diagnosed. Recently, quantitation of such mutations has also gained interest and it is expected to be of high importance in future treatment approaches.

## Gene expression profiling of malignant melanoma and development of a platform to determine and validate prognostic genes

Multiple gene expression-based prognostic biomarkers have been repeatedly identified in a variety of cancer types. Without confirmation from independent validation studies, however, the clinical utility of such biomarkers has been limited. We have previously established robust databases that enable the validation of cancer survival biomarker candidates (Gyorffy et al. 2013; Gyorffy et al. 2010; Gyorffy and Schafer 2009; Szasz et al. 2016).

**Fig. 3:**
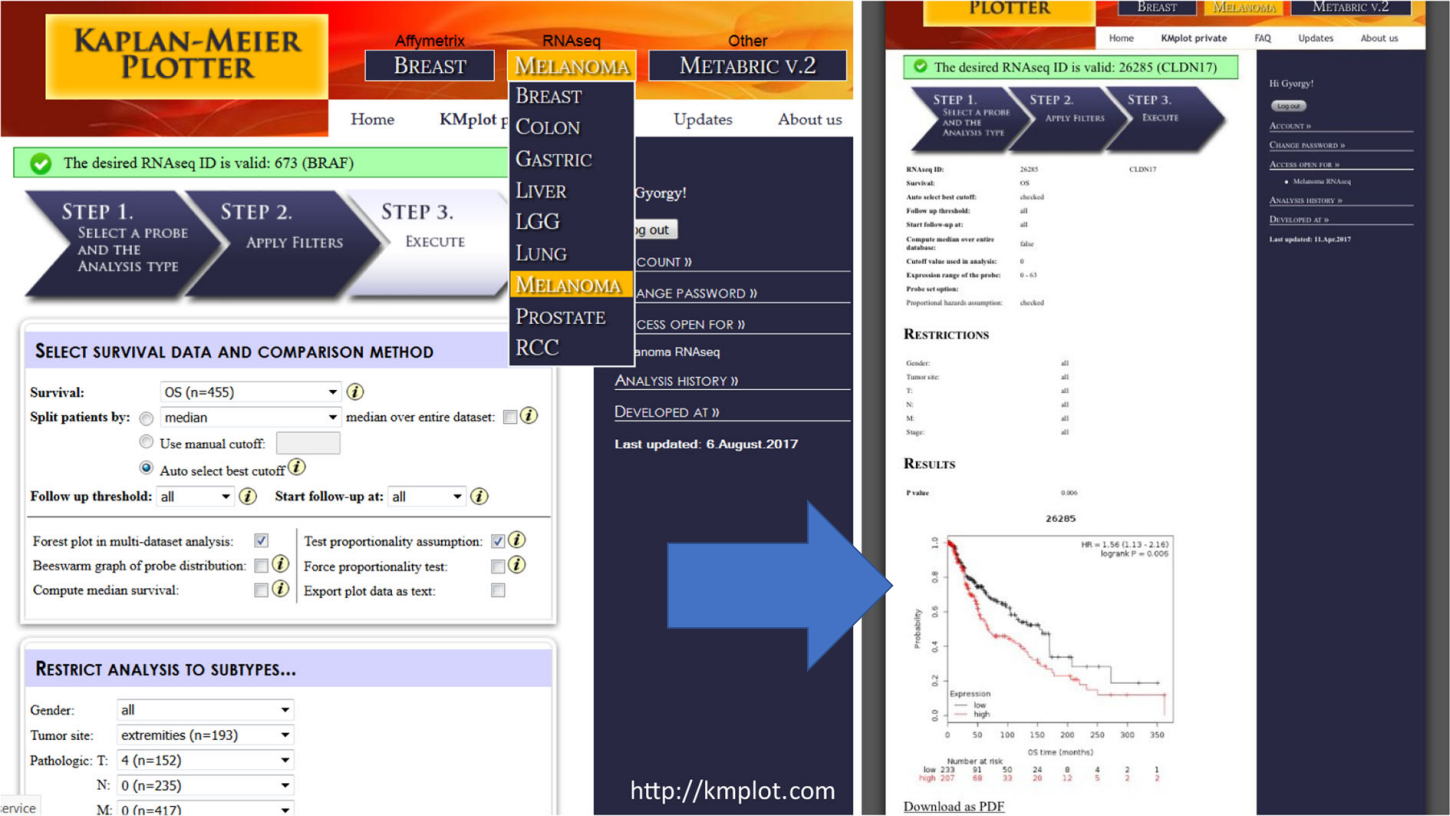
Integrated surface of the user interface of the developed platform for validation of gene expression-based biomarkers. Affymetrix chip and RNA-Seq data have been processed and annotated with clinicopathological information to provide a readily accessible and versatile tool for validation. For malignant melanoma, the TCGA data was analyzed and publicly released for non-informaticians

Herein, we integrated samples with general follow-up information, and also extended the tool towards malignant melanoma through the available RNA-Seq data of the Cancer Genome Atlas Research Network (TCGA) (The Cancer Genome Atlas Network 2015). With rapid adjustment for, e.g., gender, tumor site, and pTNM; this tool enabled validation of the prognostic information of genes from 455 patients with malignant melanoma (Fig. 3).

## Protein expression profiling melanoma heterogeneity by proteomics

Mass spectrometry-based proteomics has been extensively used to study the molecular mechanisms of cancer, to define molecular subtypes of tumors, and to map cancer-associated protein interaction networks and post-translational modifications (PTMs). Ultimately aiding the development of new therapeutics and new diagnostic and prognostic tests through the identification of cancer biomarkers (Timms et al. 2016). To date, profiling of cancer tissues have largely employed the so-called bottom-up proteomics, where the protein sample is digested (typically with trypsin) into constituent peptides prior to LC-MS/MS analysis. Improvements in speed, sensitivity, mass accuracy, and resolution of current MS instrumentation together with extensive fractionation of peptides have enabled deep coverage of cancer proteomes (Altelaar and Heck 2012; Cox and Mann 2011; Mertins et al. 2016; Smith and Kelleher 2013).

Mutations that stem from genetic alterations occur as amino acid variants in proteins translated from mRNA. In addition, many proteins are correspondingly subjected to a wide diversity of chemical modifications, i.e., PTMs such as phosphorylation and glycosylation. Many of these PTMs are linked to the function of the protein.

Integrating protein expression data with PTM data opens the possibility to verify whether the regulation occurs at the protein modification and/or at the protein abundance level. In most cases, phosphorylation is the most commonly studied PTM. Enzymes and structural proteins are involved in the process of cell signaling that is a key function linked to cancer proliferation and tumor growth.

**Fig. 4:**
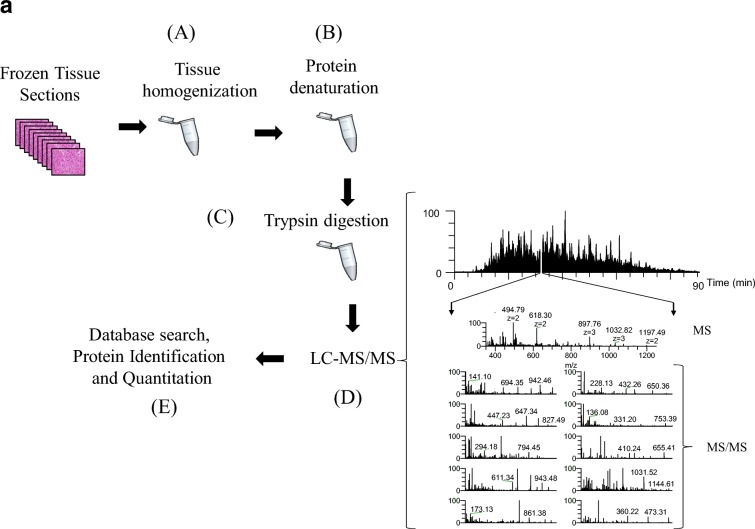

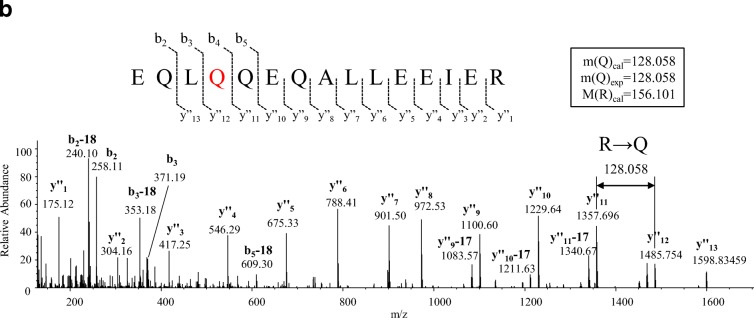
**a** The gel-free proteomic approach followed in our experiments consisted of six stages: the tissue (15–20 sections) was homogenized in a lysis buffer containing chemotropic agents such as urea or detergents (**a**); extensive denaturation of the protein extract via reduction of disulfide bridges and alkylation of free cysteine residues (**b**); proteins were enzymatically degraded to peptides with trypsin (**c**); after purification, the peptide mixture was injected onto a reversed-phase HPLC capillary column connected to a mass spectrometer and the peptides were analyzed by LC-MS/MS (**d**). Here, a mass spectrum (MS) is acquired for every peptide eluting from the LC system. The most intense peptide (precursor) ions are isolated and fragmented by collision with a neutral gas (such as Ar, He, or N_2_). This causes the peptides to dissociate into product fragment ions. At this point, a second mass spectrum (MS/MS) is recorded for the fragment ions. These two selection processes of the precursor and product fragment ions produce, highly selective mass analysis of the peptides is produced; the MS and MS/MS spectra are stored for matching against a protein sequence database using software such as SEQUEST, Mascot, and X!Tandem (**e**). The outcome of the database search is the identification of the peptides and ultimately the proteins comprising the purified protein population. In relative quantitative experiments, protein abundances are inferred from the identified peptides using dedicated software tools. **b** MS/MS of the peptide EQLQ1386QEQALLEEIER corresponding to the human plectin protein, clearly confirming the occurrence of the R1386Q mutation R (arginine) → Q (glutamine) at position 1386 of the amino acid sequence. The designation for the fragment ion signals is according to the Roepstorff–Fohlmann–Biemann nomenclature

Recently, we performed a gel-free proteomic study on regional lymph-node metastatic melanomas (Welinder et al. 2017). The samples were sectioned into 10-mm slices and subjected to histopathological examination. Each was characterized in terms of tumor, lymph-node area, necrosis, and connective tissue percentages among other parameters. MM tumors where then homogenized and analyzed by mass spectrometry (Fig. 4a). Among the tumors, 5000 proteins with a huge variation in relative quantities were identified (unpublished results).

Moreover, single amino acid variations (SAAVs) were observed in a significant number of proteins. An example of one of our findings in the MM sample cohort is depicted in the MS/MS of the peptide EQL(R1386Q)QEQALLEEIER (Fig. 4b). This peptide corresponds to the human plectin protein. In 50% of the MM tumors analyzed, however, the expected arginine residue at position 1386 was replaced with a glutamine residue. Mutations in plectin have been associated with diseases such as epidermolysis bullosa simplex with muscular dystrophy and limb-girdle muscular dystrophy (LGMD). Plectin has also been proposed as a biomarker for pancreatic cancer and esophageal squamous cell carcinoma (Bausch et al. 2011; Gundesli et al. 2010; Pawar et al. 2011).

The extreme variability displayed in the protein profiles plus the detection of missense mutations such as in the example described above confirmed the complexity and heterogeneity of the disease at the molecular level. This will deserve further comprehensive and correlation studies. By utilizing novel disease biomarkers for diagnostics and/or prognostic prediction of metastatic melanoma, the combined analysis of clinical, histological, and proteomic data should provide more personalized medicine for the patient.

## MS imaging analysis of metabolites in malignant melanoma tissue

In mass spectrometry imaging (MSI), data are systematically acquired in an array format that enables the mapping of selected ion signals by plotting ion intensity as a function of tissue position (Reyzer et al. 2003). The detection of the different endogenous or exogenous molecules is based on measuring characteristic mass-to-charge ratios (m/z); therefore, providing high selectivity. Depending on the instrumentation used, the technique also offers high spatial resolution (to the cellular level). Different ionization modes are available, but matrix-assisted laser desorption/ionization (MALDI) is perhaps the most widespread for imaging applications. In MALDI, a so-called matrix compound is applied to the samples. The matrix absorbs the energy from the laser, which is transferred to the analyte via a process referred to as “soft” ionization. MALDI-MSI is widely used to characterize drug distribution in various tissue types (Buck et al. 2015; Fehniger et al. 2011; Marko-Varga et al. 2011; Sun and Walch 2013; Torok et al. 2017; Torok et al. 2015). The method is also used to investigate various endogenous molecules, such as lipids, carbohydrates, peptides, and proteins (reviewed in (Cillero-Pastor and Heeren 2014; Gode and Volmer 2013; Harvey 2006)). MSI has gained significant interest over the past few decades from the pharmaceutical community (Nilsson et al. 2010; Swales et al. 2014). As a result of continued technical development, MSI will undoubtedly become increasingly important in pathology and in the clinic.

**Fig. 5:**
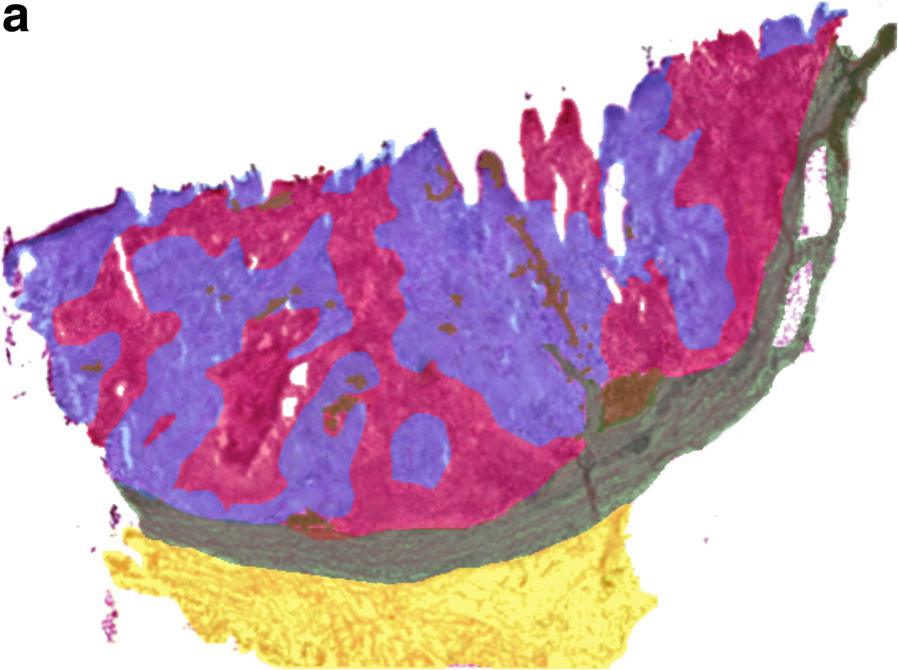

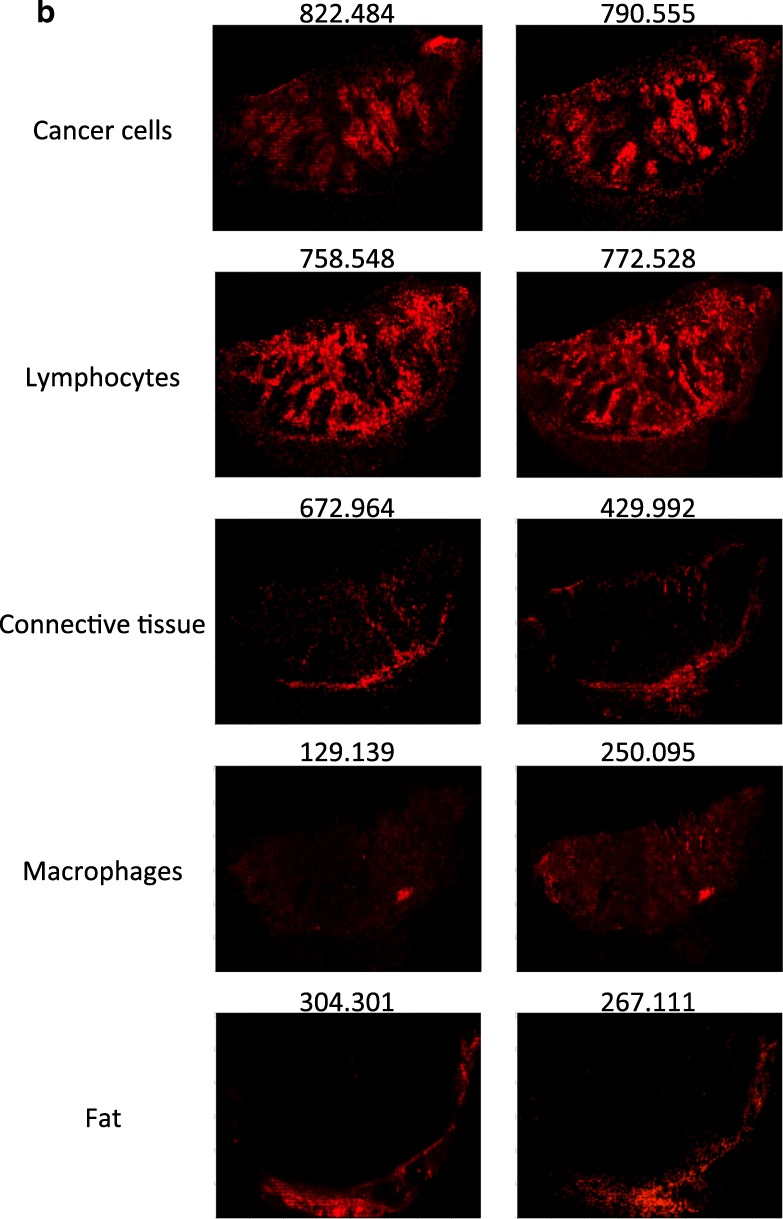

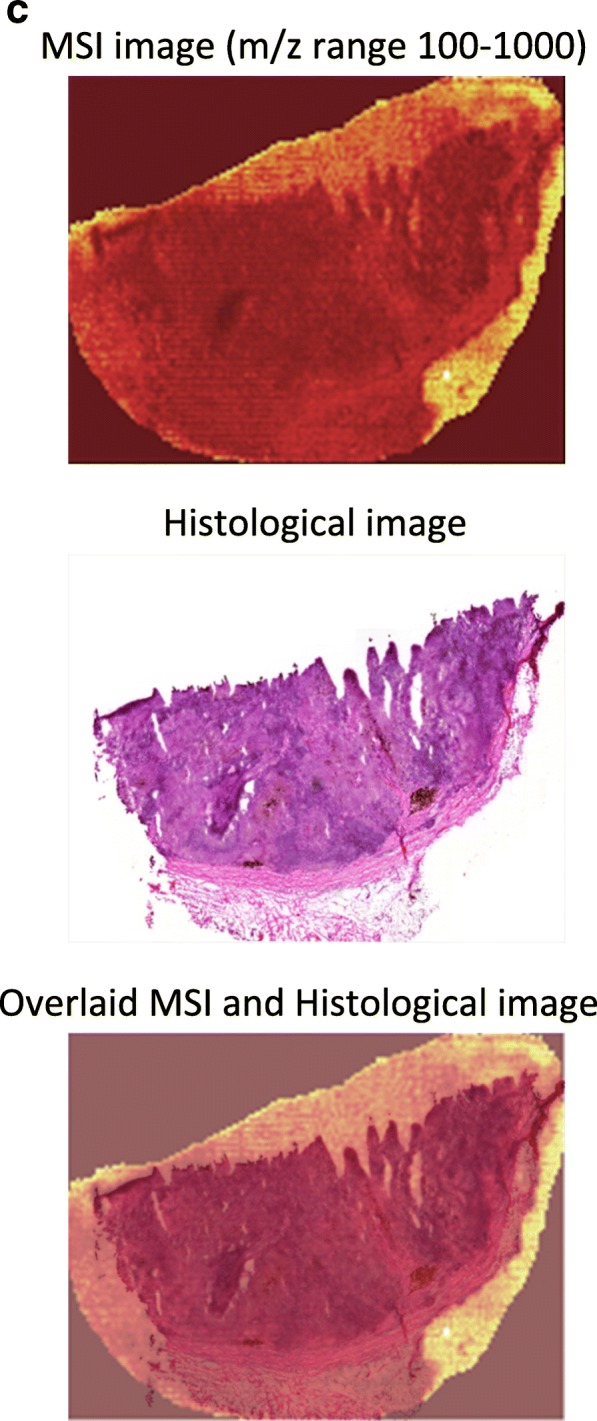
**a** H&E image of an isolated patient tumor section. Each tissue compartment is represented by a different color. Melanoma cells (area 1, purple), lymphocytes (area 2, magenta), macrophages (area 3, brown), connective tissue (area 4, green), and fat (area 5, yellow). **b** MALDI-MS images from patient tumor tissue isolated after surgery. Endogenous low-molecular weight compounds were analyzed. Images show the tissue distribution of selected masses that were correlated with various tissue compartments, such as melanoma cells, lymphocytes, macrophages, connective tissue, and fat. **c** A specific mask plane was used to query correlating masses for each investigated region. H&E stained tissue (**a**), MALDI-MSI data (m/z range 100–1000) from the same tissue slide (**b**) and the overlaid MSI and histological images (**c**)

To analyze the inherent heterogeneity in several cancer tissues, we combined MALDI-MSI with histological characterization. MALDI-MSI was performed on fresh-frozen tissue sections of MM lymph-node metastases, and low-molecular weight endogenous compounds were screened in the mass range between m/z 100–1000. After H&E staining, the same tissue section was histologically characterized. The pathologists identified several tissue compartments and cell types in the analyzed tissue samples, i.e., cancer cells, lymphocytes, macrophages, and connective tissue. Shown in Fig. 5a is an H&E stained section of a melanoma proliferative lesion and attached subcutaneous tissue. Dermal involvement of atypical melanocytes with cytologic atypia was observed. The tumor could be divided into numerous areas by the morphological features of the melanocytes. Area 1 contained large melanocytes with abundant cytoplasm and polygonal nuclei. Area 2 contained small cells with minimal cytoplasm and small nuclei composed of dense chromatin (Fig. 5b; the lymphocytic region). Area 3—infiltrated area of brown pigment-laden macrophages was also observed in the tumor and the attached subcutaneous tissue (area 4). This can also be seen in the macrophage compartment of Fig. 5c.

Once the histopathological evaluation was performed, the areas of the different tissue compartments were manually outlined to create a mask plane for each cell type. These mask planes were then used to query specific ion signatures that are correlated or anti-correlated with a given histological structure or cell type as was previously described (Fehniger et al. 2014). We observed several ion peaks with spatial distributions that correlated well with the tissue distribution of the different cell types. Some representative MALDI-MS images are illustrated in Fig. 5b. The precise identification of these characteristic masses was not attempted. Rather, rudimentary identification of the peaks was performed based on the accurate mass determined by a high-resolution Orbitrap MS instrument combined with a protein database search.

**Fig. 6:**
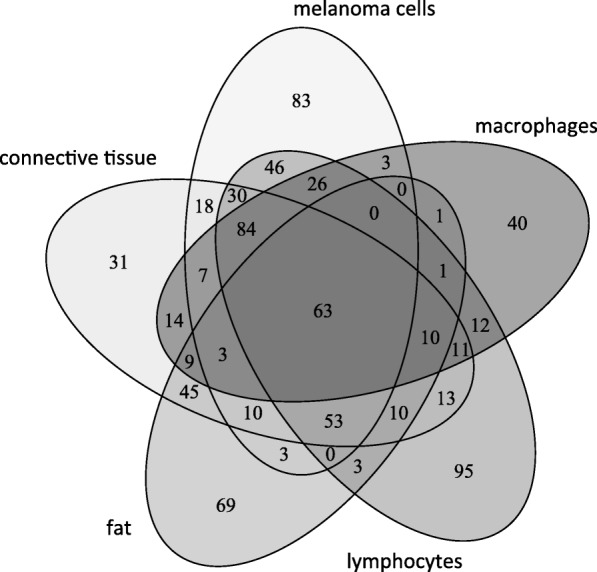
Venn diagram showing the distribution of the detected endogenous signals among the five identified tissue regions (cell types). The top 200 m/z values were extracted and used for the comparison of each representative mass spectrum

**Fig. 7:**
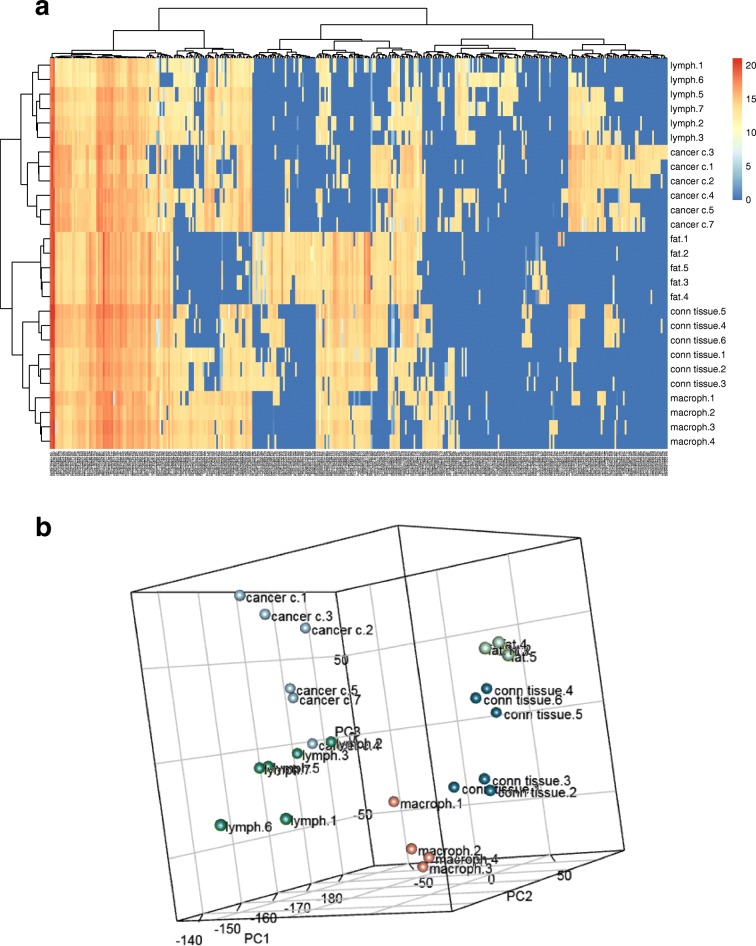
Unsupervised clustering (**a**) and PCA analysis (**b**) of the mass lists obtained from 4 to 6 representative regions of the five identified cell types

Using specific landmarks, the histological and MS images were carefully superimposed as shown in Fig. 5c. We visually assigned the borders of known histological compartments in the H&E stained samples. Then, representative spectra and peak lists (considering only the top 200 m/z values from each spectrum) of selected areas of interest were generated. The MALDIViz application was used to perform a comprehensive statistical analysis of the various peak lists (Jagadeesan and Ekstrom 2017). Multiple peaks were exclusively observed in specific tissue areas, but a large proportion of the signals showed a more general distribution within the tissue, represented as a Venn diagram (Fig. 6). After performing unsupervised clustering, most of the spectra originating from the same tissue compartment cluster together (Fig. 7).

In agreement with earlier reports (Ly et al. 2016; Meding et al. 2012), this feasibility study, which relates to the direct measurement of endogenous low-molecular weight compounds, also underlines the suitability of the MSI technique to investigate tumor heterogeneity. MALDI-MSI is appropriate for tumor phenotyping and biomarker discovery and may provide information concerning diagnosis and prognosis (reviewed in Kriegsmann et al. 2015; Norris and Caprioli 2013; Schone et al. 2013). This is of considerable value and importance as precision medicine treatments are currently rapidly developing as a first-line therapy for many cancer types. MALDI-MSI assays that can identify multiple signals from endogenous and/or therapeutic compounds in melanoma tumor tissues will provide invaluable information on the distribution and pharmacokinetic properties of pharmaceutical compounds. Subsequently, this information will be readily linked to a specific disease presentation in development and within the clinic (Sugihara et al. 2014).

## Monitoring disease and clinical decision making

Although clinical chemistry as a discipline has markedly improved, it is clear that screening cancer is still a challenging task for any healthcare organization. By using reference standards that enables normalization and equivalent disease diagnostics, central laboratories in any hospital utilize assay platforms that are globally comparable.

An aging population has increased the demand for diagnostic tests to identify disease and evaluate the quality of treatment. Laboratory-based test results are of key importance in most clinical decisions. Hospital laboratories receive samples for analysis from hospitalized patients, from family physicians, from clinical research sites, and other health clinics. The laboratory collaborates with clinicians to provide information concerning, and access to, the latest testing and treatment guidelines. Analytical methods are standardized and the laboratories participate in external quality assurance programs to ensure that the test results from different laboratories are comparable.

New knowledge gained from research in the fields of genomics and proteomics have provided new biomarkers. These are not merely diagnostic, but also prognostic and theragnostic. The link between genomics and proteomics to imaging information from microscopy-based diagnostics and radiology departments is expected to accelerate the implementation of laboratory medicine-based information in clinical practice. Future computer algorithms may extract clinically useful information from a multitude of less specific biomarkers rather than depending on the use of a single specific biomarker of which only few are currently available.

## Future directives by national health agencies

Globally, these outlined shortcomings are well known to governments and healthcare agencies. In Europe, the European Commission has dedicated large-scale research programs to address the development of disease mechanism research within dedicated research areas (Andrejevs et al. 2009). The National Institutes of Health in the USA and other sponsoring bodies in the world have also followed suit. In a joint effort between Europe and the USA, common strategies on how systems biology can be beneficial in cancer research have developed.

The pharmacogenomics area has been given industrial guidelines to use upon submission of data to the US Food and Drug Administration (http://www.fda.gov/downloads/RegulatoryInformation/Guidances/ucm126957.pdf). This document is an important milestone and a collaborative effort between the US Department of Health and Human Services, the FDA, the Center for Drug Evaluation and Research (CDER), the Center for Biologics Evaluation and Research (CBER), and the Center for Devices and Radiological Health (CDRH). A similar guideline is expected in the not too distant future that will regulate the data quality and format required for use in drug and clinical biomarker and diagnostic developments (Kudoh et al. 2008; Press et al. 1994; Slamon et al. 1989).
